# Data publication consensus and controversies

**DOI:** 10.12688/f1000research.3979.3

**Published:** 2014-10-16

**Authors:** John Kratz, Carly Strasser

**Affiliations:** 1 California Digital Library, University of California Office of the President, Oakland, CA, 94612, USA

## Abstract

The movement to bring datasets into the scholarly record as first class research products (validated, preserved, cited, and credited) has been inching forward for some time, but now the pace is quickening. As data publication venues proliferate, significant debate continues over formats, processes, and terminology. Here, we present an overview of data publication initiatives underway and the current conversation, highlighting points of consensus and issues still in contention. Data publication implementations differ in a variety of factors, including the kind of documentation, the location of the documentation relative to the data, and how the data is validated. Publishers may present data as supplemental material to a journal article, with a descriptive “data paper,” or independently. Complicating the situation, different initiatives and communities use the same terms to refer to distinct but overlapping concepts. For instance, the term *published *means that the data is publicly available and citable to virtually everyone, but it may or may not imply that the data has been peer-reviewed. In turn, what is meant by data peer review is far from defined; standards and processes encompass the full range employed in reviewing the literature, plus some novel variations. Basic data citation is a point of consensus, but the general agreement on the core elements of a dataset citation frays if the data is dynamic or part of a larger set. Even as data publication is being defined, some are looking past publication to other metaphors, notably “data as software,” for solutions to the more stubborn problems.

## What does data publication mean?

The idea that researchers should share data to advance knowledge and promote the common good is an old one, but in recent years the conversation has shifted from sharing data to *publishing* data ^1– 3^. This shift in language stems from the conviction that datasets should join the scholarly record and be afforded the same first-class status as traditional research products like journal articles ^4, 5^. While many in the scholarly communication community share this goal, different people and organizations often refer to different things with the phrase *data publication*.

Lawrence *et al.* (2011) define formal data Publication (upper-case “P”) as making data as permanently available as possible following “a process which means it can appear along with easily digestible information as to its trustworthiness, reliability, format and content” ^3^. Callaghan *et al.* (2012) draw an explicit distinction between Published and published data: published data is at least available, while Published data is persistent, documented, and peer-reviewed ^5^. Publication refers to the scholarly literature, while publication is used in the sense of any kind of printed and distributed material. Actual usage is considerably more complicated. *Data publication* overlaps with terms like *data sharing*, *data release*, and *open data*. A data publication might be a spreadsheet on a website, a set of images in an institutional archive, a stream of readings from a weather station transmitted over the internet, or a peer-reviewed article describing a dataset; a data publisher might be a data journal publisher, archive, database, or repository.

**Figure 1.**
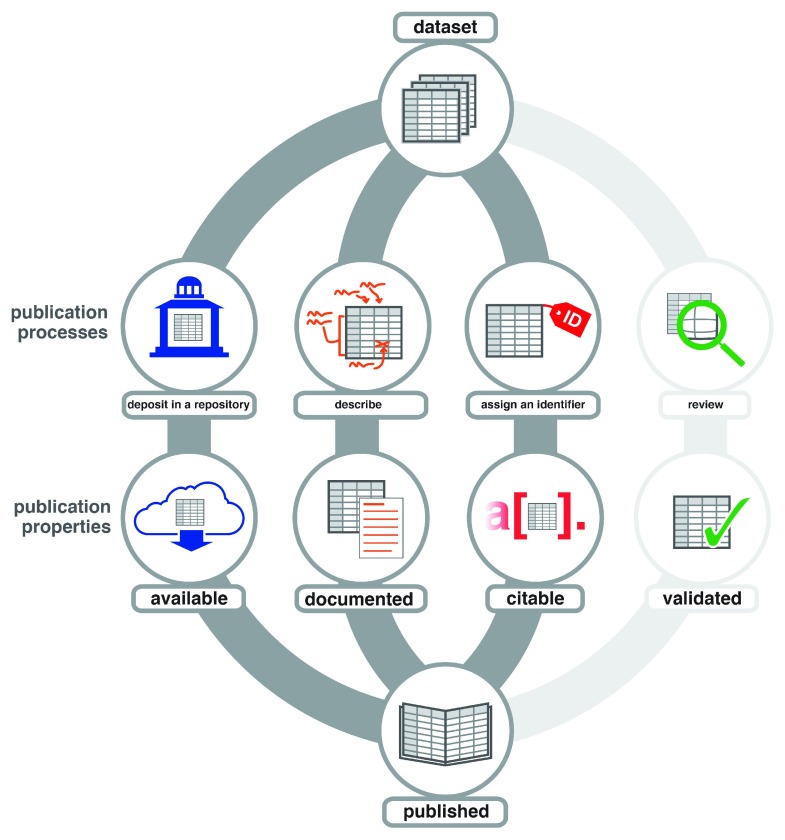
To be published, datasets are typically deposited in a repository to make them available, documented to support reproduction and reuse, and assigned an identifier to facilitate citation. Some, but not all, publishers review datasets to validate them.

Despite uncertainty over precisely what qualifies, the scholarly communication community largely agrees on three essential properties of a data publication ( Figure 1) ^2, 5^. First, published data is publicly **available** now and for the indefinite future; access might demand payment of fees or acceptance of a legal agreement, but is not subject to the whims of the author. Second, published data must be adequately **documented** such that, at a minimum, a researcher in the same field could reproduce or reuse it. Third, like a book or journal article, a data publication can be formally **cited**. Data citation maintains the integrity of the expanded scholarly record and offers a reward– in the currency of academic prestige– to encourage researchers to publish data. Open questions flock around a fourth property: how and to what extent a published dataset must be **validated**. Here, we will consider data that is persistently available, documented, and citable to be published, whatever the level of validation.

## Why publish data?

The underlying goals of data publication are to enable research to be **reproduced** and data to be **reused**. Hidden primary data exacerbates science’s very public “reproducibility crisis” ^6– 10^, recently illustrated by the collapse of a pair of irreproducible *Nature* articles describing a simple method to transform any cell into a stem cell ^11, 12^. Psychology’s “closed data culture” ^13^ enabled Diederik Stapel to invent data for an astonishing 55 papers, prompting calls for routine psychology data publication ^13– 15^. Widespread publication of the data underlying research papers could help expose honest errors as well as fraud ^16^. The leaders of the US National Institutes of Health (NIH) recently suggested “greater transparency of the data that are the basis of published manuscripts” as one way to improve scientific reproducibility ^17^.

Journals already frequently require authors to supply underlying data on request. In 2011, Alsheikh-Ali *et al.* found that 88% of high-impact journals required a statement regarding the availability of underlying data, and half of those made willingness to provide data a condition of publication ^18^. However, authors of 59% of the papers examined in the study failed to adhere to the availability instructions. Vines *et al.* (2014) could only obtain underlying data from 101 of 516 papers published from 1991 to 2011 ^19^. Availability dropped off sharply with time; out of the 62 oldest papers, data was available from only two. Now, some journals require that underlying data be published simultaneously with the article. In 2010, a coalition of Ecology and Evolutionary Biology journals began to require that the data underlying articles be archived with a maximum embargo of one year ^20, 21^. *F1000Research* has had a similar policy (without an embargo period) since its inception, and the *Public Library of Science (PLOS)* journals followed suit earlier this year ^22^.

Although there can be no substitute for funding new experiments and data collection, appropriate data reuse lowers costs and accelerates research. Documenting, publishing, and archiving data is time consuming and costly, but usually far less so than repeating the data collection. Open Context published archaeological data from a site in eastern Turkey at the substantial cost of $10,000–15,000, but this expense is minor compared to $800,000 spent to collect the data ^23^. Piwowar (2011) contrasted the impact of $100,000 in National Science Foundation (NSF) grants, which generates an average of three to four papers, with an estimate that the same investment in curating, archiving, and publishing data could contribute to over 1,000 publications ^24^. Furthermore, while some data is merely expensive to replace, time-dependent or ephemeral data, (e.g., climate records or observations of unique astronomical events) can never be recreated for any price ^25^.

## Availability

Fundamentally, to publish is to make public, and to publish data is to make data publicly available. Present availability requires mechanisms for access; future availability also requires preservation (e.g., long-term storage, format migration) ^25– 27^. As in print publication, published data need not be free or legally unencumbered, and data use agreements constrain many published datasets. If access is limited, it should be contingent on clear and objective criteria; writing a request to the creator for permission should not be part of the process. For example, before granting access to restricted data, The interuniversity Consortium for Political and Social Research (ICPSR) evaluates the applicant’s ability to handle the data securely, but not the merit of the research. The most common source of access restrictions is the need to protect the privacy of human research subjects. In the United States, the Health Insurance Portability and Accountability Act of 1996 (HIPAA) Privacy Rule severely limits the disclosure of medical information ^28^.

As a practical matter, publishing a dataset usually includes depositing it in a trustworthy repository. What constitutes “trustworthy” is somewhat subjective and there are a handful of certification schemes to choose from. In 2007, The Center for Research Libraries (CRL) published the most extensive scheme: the Trusted Repository Audit Checklist (TRAC) ^29^. Many repositories consult TRAC for self-assessment, but only four ( listed by the CRL) have completed the lengthy and rigorous process to be officially certified. That same year, DANS released the Data Seal of Approval (DSA) guidelines; 31 repositories have been stamped with the DSA since then. The Trusted Digital Repository framework incorporates the DSA, a TRAC-derived standard, and a third standard from the German Institute for Standardization (DIN) to give repositories flexibility in the processes and standards by which they are to be certified. Repositories seeking to join the World Data System (WDS) are certified to perform particular role (i.e., data publisher) based on a self-description and possibly a site visit; the WDS currently boasts 56 regular members.

Even taken together, these standards certify only a fraction of the hundreds of repositories in operation (e.g., the 973 now listed Databib or the 609 at re3data.org). In practice, the perceived trustworthiness of a repository often derives from the reputation of its managing organization. For instance, repositories run by governments or large universities are likely to be considered trustworthy (although the effects of the 2013 US government shutdown on the PubMed biomedical article database ^30^ might give one pause).

## Documentation

To be useful or reproducible, a dataset must be accompanied by descriptive information (i.e., metadata) ^25^. Preparing documentation is frequently the most laborious step for researchers in taking data from useable within the lab to useable by others, and rewarding this effort is a major impetus for data publication. Dataset documentation– which might resemble a paper– is a natural hook for bringing data into the scholarly record. The Opportunities for Data Exchange (ODE) project elaborated Jim Gray’s pyramidal model of online scientific data ^31^ into five classes of relationship between data and the literature: ‘desk-drawer’ data and four forms of publication ^4^. Similarly, five classes of data publication described by Lawrence *et al.* (2011) have recognizably different kinds of documentation ^3^. Note, however, that a single dataset may have relationships with multiple articles or other documentation, and an article may use or describe multiple datasets. Here, we will discuss three non-mutually-exclusive relationships with the literature: a dataset may **supplement** a traditional research paper, be the **subject** of a “data paper”, or be **independently documented** by its publisher.

### Data that supplements a paper

The most familiar kind of data publication is a traditional journal article accompanied by underlying data. That data can be hosted by the journal as supplementary material or deposited in a third-party repository. The trend is away from supplemental material because repositories are considered to be better suited to ensure long-term preservation and access to the data. For instance, *The Journal of Neuroscience* stopped publishing supplemental material in 2010; the announcement promotes disciplinary repositories as “vastly superior to supplemental material as a mechanism for disseminating data” ^32^. Data underlying any peer-reviewed or otherwise “reputable” publication can be deposited in the Dryad repository. Dryad makes data available and citable, but the publisher of the article must manage any assessment of scientific validity. Research Compendia compiles published articles together with all the underlying code and data. Beyond repositories like these specifically for paper-related data, many more publishers that do not require such a relationship are nevertheless pleased to publish data underlying or described by a paper.

This kind of data publication supports reproduction of an analysis, but not necessarily reuse. For example, the *PLOS* data policy requires publication of only the data needed to reproduce the article’s finding. Consequently, not all of the data collected must be published, and the documentation need not support reuse for an unrelated purpose.

### Data as the subject of a paper

A **data paper** describes a dataset with thoroughly detailed rationale and collection methods, but lacks any analysis or conclusions ^33^. Data papers are flourishing as a new article type in journals such as *F1000Research*, *Internet Archaeology*, and *GigaScience*, as well as in dedicated journals like *Earth System Science Data* ^34^, *Geoscience Data Journal*, Nature Publishing Group’s *Scientific Data*, and a trio of “metajournals” from Ubiquity Press. The strength of a data paper is in providing rich documentation, which is especially useful for unique and heterogeneous “long-tail” ^35^ research data.

Data paper length and structure varies between journals, but the tendency is toward a short, tightly structured format. All journals require an abstract, collection methods, and a description of the dataset; a few encourage authors to suggest potential uses for the data (e.g., *Internet Archaeology*, and *Open Health Data*). Some journals supplement this general framework with field-specific sections. (e.g., *Internet Archaeology* and the *Journal of Open Archaeology Data* each include a section for temporal and geographic scope). Data papers are most sharply defined not by the presence of any particular information, but by the absence of analysis or conclusions. A crisp distinction from other article types is important because many journals do not consider a data paper to be prior publication if the authors seek to publish an analysis of the same dataset (e.g., *Nature*-titled journals, *Science*, and others listed by *F1000Research*).

Data journals generally limit themselves to publishing the description of the dataset; a trusted repository publishes the data itself. For instance, *Scientific Data* and *Geoscience Data Journal* each direct authors to a list of approved repositories. One exception, *GigaScience* hosts data in an integrated repository named *GigaDB*. Another, *The International Journal of Robotics Research* ^33^ permits authors to host datasets on their own websites.

Data papers are predated by an approach that Lawrence *et al.* (2011) call *data publication by proxy*, in which a paper providing a general description of a database or dataset serves as a citable proxy ^3^. Proxy publications are distinguished from data papers in that they may contain analysis or conclusions drawn from the dataset and they may not contain all of the information needed to use the data. For example, the Climatic Research Unit of the University of East Anglia asks dataset users cite to papers associated with a dataset instead of the data itself. In the biosciences, *Nucleic Acids Research (NAR)* annually publishes a massive issue devoted to such articles; the 2014 database issue featured 58 papers describing new databases and 123 updates on existing resources ^36^. Participating databases typically ask users to cite the most recent *NAR* paper. Proxy publication or data paper citation serves to award scholarly credit, but fails at other functions of citation and should be supplemented with direct citation of the data.

### Independent documentation

Dataset documentation need not take the form of a journal article. Together with data, repositories and databases publish documentation– minimal or rich, structured or freeform– that sometimes fulfills the needs of reproducibility and reuse without reference to the literature. Even so, an independently documented dataset might also be described by a data paper or support any number of traditional articles. Academic, governmental, and commercial repositories publish data from diverse place- and interest-based research communities through varying processes with or without linkage to the literature.

Institutional repositories preserve and publish any kind of data generated by the research communities they serve, e.g., University of California researchers deposit data in Merritt, while Purdue University researchers use the Purdue Research Repository (PURR). At the national level, the Dutch Data Archiving and Networked Services (DANS) accepts a broad range of data from researchers in the Netherlands. Figshare and Zenodo publish data from any researcher in any field. These broad-topic publishers are well suited to handle heterogeneous or long-tail data that does not fit comfortably in a specialized repository. But, because repositories typically cannot assemble domain expertise across such a broad range of disciplines, this inclusiveness imposes limits on documentation requirements and validation. While Figshare and Zenodo do accommodate rich documentation, they require very little.

Interest-based research communities are served by a thriving ecosystem of specialized data publishers. The broadest of these publishers serve entire disciplines, e.g., the Digital Archaeological Record (tDAR). A narrower example from the life sciences is the group of databases centered around model organisms, such as WormBase ^37^ or FlyBase ^38^; these databases aggregate diverse, but finite, data types and benefit from extensive domain expertise. Along similar lines, a data publisher may deal with a particular data-type, such as gene expression data in the Gene Expression Omnibus (GEO) or seismological data in SeismicPortal. Focus on a particular type of data facilitates rigorous technical validation and development of specialized metadata requirements to ensure the data is useable. For instance, GEO data ingest meshes with Minimum Information About a Microarray Experiment (MIAME) ^39^ documentation guidelines ^40^. As a final example, a publisher might be devoted to a particular scientific instrument or facility, such as the One Degree Imager Portal, Pipeline, and Archive (ODI-PPA) or the Worldwide LHC Computing Grid), the massive infrastructure built to handle the output of the Large Hadron Collider (LHC). Unlike most other publishers, these emphasize real time access to data coming off the instruments.

Because researchers know the databases that serve their community, data in disciplinary repositories is easy to discover and because it is relatively standardized, it is easy to reuse. A disadvantage is that the data from a single research program can be distributed across many repositories (e.g., gene expression data in one, sequence data in another), whereas an institutional or broad-scope repository can publish the whole research story.

## Citability

Data citation is the element of publication that has come the farthest toward consensus. In early 2014, a coalition of organizations brought together by Future Of Research Communication and E-Scholarship (FORCE11) ^41^ released a Joint Declaration of Data Citation Principles. The first of the eight principles states, in part, that “[d]ata citations should be accorded the same importance in the scholarly record as citations of other research objects, such as publications”. Most of the time, this means that when a published dataset contributes to a paper, it should be cited formally in the reference list.

Unfortunately, actual practice lags far behind this consensus. Not all article publishers allow data citations in the references and, even when permitted, most authors refer to data in the text without a formal citation ^42^. Many data publishers provide no guidance on citation; others ask users to cite a proxy publication (e.g., from the *NAR* database issue). However, a growing number of data publishers do supply users with explicit citation instructions; Dryad, Figshare, and Zenodo dataset landing pages all display a formatted citation and links for import into reference managers.

Many data publishers facilitate formal citation by assigning unique permanent identifiers, most commonly the same ones used for journal articles: Digital Object Identifiers (DOIs). In addition to precisely specifying what resource is being cited, a DOI can be resolved to locate the referenced dataset. Note, however, that a DOI is neither sufficient nor necessary for citability, which demands that the referenced object be persistent and locatable via the citation. If a dataset moves and the DOI is not updated with the new location, the citation breaks. Conversely, a well-maintained web-address works as well as a DOI in theory– although a DOI is more likely to be maintained in practice.

### Simple case

The present consensus is that a dataset should be cited using, at a minimum, five elements largely familiar from article citations: creator(s), title, year, publisher and identifier. This format agrees with Committee on Data for Science and Technology (CODATA) recommendations ^43^ and conveys all the information required to obtain a DataCite DOI ^44^ or be listed in the Thomson-Reuters Data Citation Index. The basic format works well when a dataset can be cited like an article, but that is not always the case.

### Deep citation

One major complication data citation faces is the need for **deep citation**. When supporting an assertion in writing, it usually suffices to cite the entirety of an article or the page of a book and leave it to the inquisitive reader to find the relevant passage. But, to reproduce an analysis performed on a subset of a larger dataset, the reader needs to know exactly what subset was used (e.g., a limited range of dates, only the adult subjects, wind speed but not direction). Datasets vary so widely in structure that there may not be a good general solution for describing subsets. The most common suggestion is to cite the entire dataset in the reference list and describe the subset in the text of the paper ^45^. The Federation of Earth Science Information Partners (ESIP) and the National Snow and Ice Data Center (NSIDC) both recommend defining the subset in the citation itself, using a format suited to the dataset’s internal structure (e.g., a temporal or spatial range, a list of variables, or an internal identifier).

### Dynamic datasets

A second major complication arises when datasets change. In the past, the printing process cemented one version of an article as the version of record. Even for traditional scholarly literature, web-based publishing and preprint servers (e.g., arXiv.org) are complicating the situation, but datasets are especially prone to be **dynamic**. Two kinds of dynamic datasets warrant consideration: **growing** datasets that add new data while never changing or deleting existing data, and **revisable** datasets where data may by added, deleted, or changed.

Consider USC00046336, a weather station at the Oakland Museum of California. Each day, the high temperature, low temperature and amount of precipitation recorded at the Museum ^46^ flow, together with data from more than 20,000 other stations, into the swelling Global Historical Climate Network (GHCN)-Daily ^47^ dataset. Or, consider WormBase, the genome database used by the *Caenorhabditis elegans* research community. WormBase encompasses genomic sequences of *C. elegans* and 20 related species massively annotated with gene structures, protein sequences, expression patterns, and a host of other information from empirical data and computational predictions. Every two months, WormBase administrators respond to new data and better computational models by issuing a revised version with new material added and inaccurate material deleted or corrected.

Additions and updates to published datasets are extremely valuable, but a researcher seeking to reproduce an analysis of a dynamic dataset needs access to a particular version. To enable that access, previous versions must be preserved and citable. Growing datasets can be cited with an access date or a date range in the citation, as recommended by ESIP and NSIDC. Revisable datasets are more difficult; the most common approach is to accumulate revisions and periodically publish a new version with a citable version number. For example, WormBase identifies each release with a version number and makes all of the previous versions available.

Controversy persists around the specific issue of identifiers for dynamic datasets. DataCite recommends, but does not insist, that their DOIs refer to immutable digital objects. NSIDC and ESIP instruct researchers to use a single identifier for growing datasets and include the access date in the citation; each major version of a revisable datasets gets a new identifier, but minor versions do not. In contrast, the Digital Curation Centre (DCC), Dataverse, and the UK Natural Environment Research Council (NERC) insist that any change to a dataset should trigger a new identifier ^5, 45, 48^. To handle the difficulties with dynamic data that this policy creates, the DCC recommends periodically issuing growing datasets a new identifier that refers to the *time-slice* of new records and freezing versions of revisable datasets as individually-identified *snapshots*.

### Just-in-time identifiers

The difficulties surrounding deep citation and dynamic data could potentially be solved by turning the identifier-issuing process on its head. Instead of the dataset publisher issuing identifiers for data at the level that researchers seem likely to cite, researchers could issue identifiers for only the part of the dataset that they want to cite.

The Research Data Alliance (RDA) Data Citation Working Group recently put forth a sophisticated proposal applicable to data in (or convertible to) databases. Identifiers created under this scheme would wrap together identification of a database, a query to return the cited dataset, the version of the database queried for this analysis, and a number of other useful components. The ultimate promise is to provide a simple yet precise citation for any selection of data, at the cost of technical complexity “under the hood”.

## Validation

Data validation is the least resolved aspect of data publication, and fundamental questions are still unanswered: What minimum level of quality should a published dataset guarantee? How and by what criteria can datasets be evaluated against that guarantee? How should dynamic datasets be handled? Is literature peer-review an appropriate model?

Callaghan *et al.* (2012) ^5^ draw a useful distinction between **technical** and **scientific** review. Technical review verifies that a dataset is complete, its description is complete, and that the two match up. Domain expertise is generally not required, and many repositories provide at least some level of technical review. Scientific review evaluates the methods of data collection, the overall plausibility of the data, and the likely reuse value. Scientific review does require domain expertise, making this level of validation more difficult to organize ^13^. When data is published with a data paper, review may be split between the repository for technical review and the data journal for scientific review.

### Data paper peer review

Peer review guarantees that journal articles entering the scholarly record reach some level of validity (although the aforementioned reproducibility crisis calls into question exactly what that level is). In many fields, peer-reviewed publications enjoy a much higher status than any other literature. Any effort to apply the prestige of “publication” to datasets cascades naturally into an effort to apply the prestige of “peer review”. But as data validation seeks to model itself on literature peer review, literature peer review itself is in flux ^49– 51^. Open peer review at *F1000Research* and post-publication commenting at PubMed Commons are just two of many ongoing web-enabled experiments in article evaluation.

Journal article reviewers traditionally consider whether the methods used are appropriate for the questions asked and the data collected support the conclusions drawn. In the absence of particular questions and conclusions, it is not obvious what peer review of data should certify. A dataset may serve for some purposes, but not for others and a reviewer may anticipate many potential uses for the data, but surely not all ^52^. Researchers are already over-whelmed by peer review of articles ^53^ and could find any increased workload unreasonable. Despite all these difficulties, venues for peer-reviewed data papers are opening rapidly.

Data paper journals wrap scientific peer review of the paper and the dataset together into a single process. *GigaScience*, an exception, assigns technical review of the dataset to a separate data reviewer. The guidelines that various data journals provide to reviewers are fairly uniform, except that about half consider novelty or potential impact, while the rest only require the dataset to be scientifically sound. Although the guidelines are similar, review processes differ widely.

As an example, compare *Biodiversity Journal* and *Scientific Data*. Both journals divide reviewer guidelines into three sections along similar lines, which *Biodiversity Journal* calls “quality of the data”, “quality of the description”, and “consistency between manuscript and data”. *Scientific Data* follows a traditional peer-review process: an editor appoints reviewers who are encouraged to remain anonymous. In contrast, review at *Biodiversity Journal* follows a flexible and open process featuring entirely optional anonymity and multiple types of reviewer. There, an editor appoints two or three “nominated” reviewers who must report back and several “panel” reviewers who read the paper and only comment at their discretion. Additionally, the authors may choose to open the paper to public comment during the review process.

### Independent data validation

Data journals all model their data validation more or less faithfully on literature peer review, but independent data validation practices and proposals are considerably more varied. Lawrence *et al.* (2011) propose a set of independent data peer review guidelines similar to the ones used by data journals ^3^. Each of The National Aeronautics and Space Administration (NASA) Distributed Active Archive Centers (DAACs) draws on an affiliated User Working Group for domain expertise. The NSIDC combines an internal assessment of the effort that will be required to publish a dataset at a desired level of service (roughly corresponding to technical review) with an external assessment of scientific quality. The Planetary Data System (PDS) peer-reviews datasets via an in-person meeting with representatives of the repository, the dataset creators, and the reviewers.

Pre-publication validation can be supplemented or replaced by post-publication feedback from successful or unsuccessful reusers. Parsons *et al.* (2010) suggest that “data use in its own right provides a form of review”, and go on to point out that the context of reuse demonstrates that the data is not simply “good”, but fit for some particular purpose ^52^. The DANS repository solicits feedback from researchers who use its datasets: users are asked to rate the dataset on a one to five scale in each of six criteria (e.g., data quality, quality of the documentation, structure of the dataset) ^54, 55^. Researchers trust peer review in part because they understand the process and its limitations; if researchers come to understand them, alternate pre- or post-publication validation processes could potentially provide the same level of assurance.

Two examples from archaeology, Open Context and the Digital Archaeological Record (tDAR), illustrate the diversity of approaches to data validation. Open Context provides multiple validation processes that incorporate peer review beyond a simple accept/reject binary ^23^. Each Open Context dataset is rated from one to five based not on quality *per se*, but on the thoroughness of the validation; a one comes with no guarantees, a three has passed a technical review, and a five has passed external peer review. Whereas Open Context is a boutique publisher, focusing on data presentation and reuse, tDAR is a large repository primarily concerned with collecting and preserving archaeology data for future use. tDAR is able to operate at scale by performing only technical validation and streamlining data deposition with a minimum of mandatory description. However, tDAR also serves as a platform for high-quality data publication. The repository accommodates contributors who provide more information, and much of the content is deposited by digital curators who can be relied on to supply rich descriptions. Furthermore, two data paper journals, *Internet Archaeology* and *Journal of Open Archaeological Data*, recommend both tDAR and Open Context as repositories for their peer-reviewed data. Thus, data validation depends not only on discipline and data type, but on a host of external factors, including the goals of the organizations and researchers involved.

## Beyond data publication

Consensus abides wherever traditional scholarly publication offers a clear model for data; controversy churns wherever the literature offers only murky guidance. Static datasets of manageable size and simple structure can be made available, identified, and cited like the literature. Dynamic and complex datasets raise questions that attract multiple and sometimes conflicting answers. Where the guidance of the print metaphor threatens to give out, it must be extended creatively— or abandoned for another approach entirely.

Parsons and Fox (2013) ^56^ argue that thinking about data through the metaphor of print publication is often misleading. They advocate treating publication as only one metaphor in a larger ecosystem of metaphors for sharing data. For example, they associate the uniform, high-volume output from instruments like the Large Hadron Collider with industrial production and suggest “Big Iron” as an alternative metaphor for this kind of data.

Another alternative metaphor that seems to be gaining particular traction is “data as software” ^57^. Here, one thinks of releasing a dataset like a piece of software and regards subsequent changes as analogous to updated versions. The open-source software community has already developed many potentially relevant tools for working collaboratively, managing multiple versions, and tracking attribution. Ram (2013) ^58^ catalogs a multitude of scientific uses for the software version control system Git, including data management. Open Context uses Git and Mantis Bug Tracker to track and correct dataset errors. The Dat project “aim[s] to bring to data a style of collaboration similar to what Git brings to source code”. Furthermore, projects such as IPython Notebook integrate data, processing, and analysis into a single package. Unfortunately, scientific software struggles for recognition ^59^ just as data does, so that metaphor offers little guidance for navigating the academic reward system. On the other hand, the publication metaphor targets this system explicitly, but leaves numerous other gaps.

Although some aspects of data publication have matured to a firm and useful consensus– exemplified most powerfully by the Joint Declaration of Data Citation Principles– the field as a whole is still burgeoning. Controversial issues, such as validation, may be best addressed by presenting an array of options rather than converging on a single solution. In the ongoing conversation, *data publication* may come to refer to only those means of dissemination most directly drawn from the scholarly literature, or it may open as a canopy over a range of approaches. Whichever the case, it is our hope and expectation that for the foreseeable future, mixing of metaphors and contemplation of the unique properties of research data will continue to yield novel forms of data-centered scholarly production.
