# Supplementary material for: Data publication consensus and controversies
Source: F1000Res. 2014 Oct 16;3:94. Originally published 2014 Apr 23. [Version 3] doi: 10.12688/f1000research.3979.3 (PMC4097345; doi:10.12688/f1000research.3979.3)
Supplement: Peer review history

# Peer review: Referee response for version 3

**DOI:** 10.5256/f1000research.5878.r6447

**Published:** 2014-11-06

**Authors:** Mark Parsons

**Affiliations:** 1 Research Data Alliance, Troy, NY, USA

A nice rewrite and a very much improved paper, especially since it is now properly classified as a review article. Just a few small corrections listed below.

Page 4, Para 5:

"Writing a request to the creator should [very rarely] be part of the process" As noted before creator permission is sometimes legitimate.

Page 5, para 2:

"The most familiar kind of data publication is a traditional journal article accompanied by underlying data. " [citation needed] or change the sentence.

Page 5, para 7

"Data papers are predated by an approach that Lawrence et al. (2011) call data publication by proxy..." This is not true. As Hans Pfeiffenberger notes, ESSD dates back to 2008. Indeed I wouldn't be surprised if Lawrence chatted a bit with ESSD editors, Pfeiffenberger and Carlson, in preparing his paper.

Page 6, para 1

NSIDC does do external scientific reviews but they only go outside when they don’t have expertise in house. So it's sort of a combined approach. A quibble, but they pride themselves on in-house scientific expertise and engagement.

Page 6 para 2:

This paragraph is a little confused. Domain repositories don’t usually serve interdisciplinary use very well, but I don’t see how it’s necessarily bad to be distributed. What do you mean by publishing the whole research story? No one entity can do that.

I have read this submission. I believe that I have an appropriate level of expertise to confirm that it is of an acceptable scientific standard.

# Peer review: Referee response for version 2

**DOI:** 10.5256/f1000research.4518.r4540

**Published:** 2014-06-10

**Authors:** Ingrid Dillo

**Affiliations:** 1 Data Archiving and Networking Services (DANS), The Hague, Netherlands

The article focuses on a topic that receives a lot of interest these days. Therefore it is very timely. The article provides a useful and valuable overview of the current state of affairs and the ongoing debate. The title does justice to the content of the article. So does the abstract.

This is the second version of the article. I do not see many changes in the text based on the earlier critical comments made by Mark Parsons and Peter Fox.

The overview is very informative for everyone who needs a quick introduction into the subject. I do miss the opinion of the authors themselves on the issues at hand and on the quoted suggestions by others. This would have been appropriate in a concluding paragraph.

**Detailed comments:**

1. In the Introduction I miss a clear link between data publishing and data citation and creating the possibility for researchers to receive academic credits for their work on data. This academic credit is crucial as an incentive for researchers to put valuable time and effort in sharing their data.
2. In the paragraph *Why publish data?* a reference to the Dutch fraud cases might be useful, as these cases got a lot of international attention and more or less triggered the discussion in the Netherlands with respect to research data management, long term preservation of data and data publishing and citation. A reference could be:
  Doorn P, Dillo I, van Horik R: Lies, Damned Lies and Research Data: Can Data Sharing Prevent Data Fraud? *International Journal of Digital Curation. *2013; **8**(1): 229-243 http://dx.doi.org/10.2218/ijdc.v8i1.256
3. In the paragraph *Types of data publication* a threefold model is introduced to categorize data publications. It is not clear how this model relates to the terminology and categorisation presented in the *introduction. *This could be somewhat confusing for the reader.
  Furthermore, there are of course many other models available, e.g. that of the The Data Publication Pyramid, developed on the basis of the Jim Gray pyramid, to express the different manifestation forms that research data can have in the publication process:
  Reilly S, Schallier W, Schrimpf S, *et al.*: Report on integration of data and publications. October 2011. Located at: http://www.stm-assoc.org/2011_12_5_ODE_Report_On_Integration_of_Data_and_Publications.pdf 
  Or the model presented in the report: Costas, R., Meijer, I., Zahedi, Z. and Wouters, P. (2013). The Value of Research Data - Metrics for datasets from a cultural and technical point of view. A Knowledge Exchange Report, available from www.knowledge-exchange.info/datametrics 
4. With respect to trustworthy digital repositories, I would like to add a few comments. First of all, in Europe a European Framework for Audit and Certification of Digital Repositories is emerging. It contains three certification standards (DSA, DIN31644/NESTOR seal and ISO13636) and three levels of certification (basic, extended and formal) see: http://www.trusteddigitalrepository.eu/Site/Trusted%20Digital%20Repository.html 
  Of these three standards, only DSA has been up and running for some time now ,with 31 seals awarded and 30 ongoing self-assessments at this moment. The NESTOR seal has become available only very recently and the ISO standard is not yet officially available. The accompanying ISO 16919 standard: Requirements for bodies providing audit and certification of candidate trustworthy digital repositories, has been published very recently and now the ISO organization needs to be set up in the different countries, including the training of national auditors. The audits done by CRL are not fully official, since CRL is no formal ISO accreditation body.
  In Europe we see a growing interest in TDRs, coming from funders who want to push open data and data sharing and demand the deposit of publicly funded data in long term TDRs. Furthermore European research infrastructures and projects are also looking more and more into the issue of trust hen it comes to data sharing and a groeing number of them is incorporating (parts of) the DSA guidelines into there repositories and policies (e.g. CESSDA, CLARIN, EUDAT).
  Yet another certification procedure is offered by the ICSU/WDS to repositories that aim to become a member of the World Data System. See: https://www.icsu-wds.org/community/membership/certification 
  The certification of TDRs could also help publishers/editorial boards with Data Availability Policies to point their authors to the right repositories for the long-term storage of their data.

I have read this submission. I believe that I have an appropriate level of expertise to confirm that it is of an acceptable scientific standard.

# Peer review: Referee response for version 2

**DOI:** 10.5256/f1000research.4518.r4543

**Published:** 2014-05-28

**Authors:** Mark Costello

**Affiliations:** 1 Institute of Marine Science, University of Auckland, Auckland, New Zealand

Having made some similar comments myself I must agree with this review. But a few points may merit amendment:

**Abstract:**

Note that what 'publication' means is the same as in print as in digital form. It does not imply peer-review or editorial oversight in either format.

**Citability:**

Yes, data citations are important but some datasets and web-based resources do not show how they should be cited, some journals do not allow citations to web resources in the Reference list, and even were both possible, too many authors neglect to cite actual datasets and instead cite a web site (which may have many datasets) or a related print paper.

I agree that a DOI is not enough and only permanent if it is updated when documents are moved. A full author-tile-publisher citation as you suggest is more informative and human readable.

I do not think it is problematic to cite parts of datasets. Pages and chapters in books are already cited for example. In most datasets it is also possible to identify individual dat records. Also, the actual data used could be provided in an Appendix so the reader is left in no doubt. Neither do I think 'versioning' is a problem. Where new data are added (e.g. to a time-series) then they comprise a new dataset, as they would if published in print. Where many corrections are made they a dataset can be treated like a paper; i.e. the original can be 'retracted' and replaced, or the new version be published with the metadata stating that it is more accurate.

You mention venues for peer-reviewed data papers. For this article to advance previous articles, perhaps it could expand on these venues and how they manage the details of the peer-review process?

I have published a few papers you may find of interest:

1. Costello MJ, Wieczorek J. 2014. Best practice for biodiversity data management and publication. *Biological Conservation,* 173, 68-73. http://www.vliz.be/en/imis?module=ref&refid=234968
2. Costello MJ, Appeltans W, Bailly N, Berendsohn WG, de Jong Y, Edwards M, Froese R, Huettmann F, Los W, Mees J, Segers H, Bisby FA. 2014. Strategies for the sustainability of online open-access biodiversity databases. *Biological Conservation* 173, 155-165. http://www.marinebiology.ugent.be/component/imis/?module=ref&refid=230520
3. Costello MJ, Michener WK, Gahegan M, Zhang Z-Q, Bourne P. 2013. Data should be published, cited and peer-reviewed. *Trends in Ecology and Evolution *28 (8) *,* 454-461. http://dx.doi.org/10.1016/j.tree.2013.05.002
4. Costello, M.J., Vanden Berghe E. 2006. “Ocean Biodiversity Informatics” enabling a new era in marine biology research and management. *Marine Ecology Progress Series* 316, 203-214. http://www.int-res.com/abstracts/meps/v316/
5. Costello MJ, Michener WK, Gahegan M, Zhang Z-Q, Bourne P, Chavan V. 2012. Quality assurance and intellectual property rights in advancing biodiversity data publications. ver. 1.0, Copenhagen: Global Biodiversity Information Facility, Pp. 33, ISBN: 87‐92020‐49‐6. Accessible at http://links.gbif.org/qa_ipr_advancing_biodiversity_data_publishing_en_v1.

I have read this submission. I believe that I have an appropriate level of expertise to confirm that it is of an acceptable scientific standard.

# Peer review: Referee response for version 1

**DOI:** 10.5256/f1000research.4264.r4541

**Published:** 2014-05-06

**Authors:** Mark Parsons, Peter Fox

**Affiliations:** 1 Research Data Alliance, Troy, NY, USA; 2 Rensselaer Polytechnic Institute, Troy, NY, USA

**General Comments**:

*Note: This review was written by Parsons and accepted (with some modification) by Fox. Insights likely come from conversations between Fox and Parsons, errors from Parsons*.

I am very glad the authors wrote this essay. It is a well-written, needed, and useful summary of the current status of “data publication” from a certain perspective. The authors, however, need to be bolder and more analytical. This is an opinion piece, yet I see little opinion. A certain view is implied by the organization of the paper and the references chosen, but they could be more explicit. The paper would be both more compelling and useful to a broad readership if the authors moved beyond providing a simple summary of the landscape and examined *why* there is controversy in some areas and then use the evidence they have compiled to suggest a path forward.  They need to be more forthright in saying what data publication means to them, or what parts of it they do not deal with. Are they satisfied with the Lawrence *et al.* definition? Do they accept the critique of Parsons and Fox? What is the scope of their essay?

The authors take a rather narrow view of data publication, which I think hinders their analyses. They describe three types of (digital) data publication: Data as a supplement to an article; data as the subject of a paper; and data independent of a paper. The first two types are relatively new and they represent very little of the data actually being published or released today. The last category, which is essentially an “other” category, is rich in its complexity and encompasses the vast majority of data released. I was disappointed that the examples of this type were only the most bare-bones (Zenodo and Figshare).  I think a deeper examination of this third category and its complexity would help the authors better characterize the current landscape and suggest paths forward.

Some questions the authors might consider: Are these really the only three models in consideration or does the publication model overstate a consensus around a certain type of data publication? Why are there different models and which approach is better for different situations? Do they have different business models or imply different social contracts? Might it also be worthy of typing “publishers” instead of “publications”? For example, do domain repositories vs. institutional repositories vs. publishers address the issues differently? Are these models sustaining models or just something to get us through the next 5-10 years while we really figure it out?

I think this oversimplification inhibited some deeper analysis in other areas as well. I would like to see more examination of the validation requirement beyond the lens of peer review, and I would like a deeper examination of incentives and credit beyond citation.

I thought the validation section of the paper was very relevant, but somewhat light. I like the choice of the term validation as more accurate than “quality” and it fits quite well with Callaghan’s useful distinction between technical and scientific review, but I think the authors overemphasize the peer-review style approach. The authors rightly argue that “peer-review” is where the publication metaphor leads us, but it may be a false path. They overstate some difficulties of peer-review (No-one looks at every data value? No, they use statistics, visualization, and other techniques.) while not fully considering who is responsible for what. We need a closer examination of different roles and who are appropriate validators (not necessarily conventional peers). The narrowly defined models of data publication may easily allow for a conventional peer-review process, but it is much more complex in the real-world “other” category. The authors discuss some of this in what they call “independent data validation,” but they don’t draw any conclusions.

Only the simplest of research data collections are validated only by the original creators. More often there are teams working together to develop experiments, sampling protocols, algorithms, etc. There are additional teams who assess, calibrate, and revise the data as they are collected and assembled. The authors discuss some of this in their examples like the PDS and tDAR, but I wish they were more analytical and offered an opinion on the way forward. Are there emerging practices or consensus in these team-based schemes? The level of service concept illustrated by Open Context may be one such area. Would formalizing or codifying some of these processes accomplish the same as peer-review or more? What is the role of the curator or data scientist in all of this? Given the authors’s backgrounds, I was surprised this role was not emphasized more. Finally, I think it is a mistake for science review to be the main way to assess reuse value. It has been shown time and again that data end up being used effectively (and valued) in ways that original experts never envisioned or even thought valid.

The discussion of data citation was good and captured the state of the art well, but again I would have liked to see some views on a way forward. Have we solved the basic problem and are now just dealing with edge cases? Is the “just-in-time identifier” the way to go? What are the implications? Will the more basic solutions work in the interim? More critically, are we overemphasizing the role of citation to provide academic credit? I was gratified that the authors referenced the Parsons and Fox paper which questions the whole data publication metaphor, but I was surprised that they only discussed the “data as software” alternative metaphor. That is a useful metaphor, but I think the ecosystem metaphor has broader acceptance. I mention this because the authors critique the software metaphor because “using it to alter or affect the academic reward system is a tricky prospect”. Yet there is little to suggest that data publication and corresponding citation alters that system either. Indeed there is little if any evidence that data publication and citation incentivize data sharing or stewardship. As Christine Borgman suggests, *we need to look more closely at who we are trying to incentivize to do what*. There is no reason to assume it follows the same model as research literature publication. It may be beyond the scope of this paper to fully examine incentive structures, but it at least needs to be acknowledged that building on the current model doesn’t seem to be working.

Finally, what is the takeaway message from this essay? It ends rather abruptly with no summary, no suggested directions or immediate challenges to overcome, no call to action, no indications of things we should stop trying, and only brief mention of alternative perspectives. What do the authors want us to take away from this paper?

Overall though, this is a timely and needed essay. It is well researched and nicely written with rich metaphor. With modifications addressing the detailed comments below and better recognizing the complexity of the current data publication landscape, this will be a worthwhile review paper. With more significant modification where the authors dig deeper into the complexities and controversies and truly grapple with their implications to suggest a way forward, this could be a very influential paper. It is possible that the definitions of “publication” and “peer-review” need not be just stretched but changed or even rejected.

**Detailed comments:**

- The whole paper needs a quick copy edit. There are a few typos, missing words, and wrong verb tenses. Note the word “data” is a plural noun. E.g., Data are not software, nor are they literature. (NSICD, instead of NSIDC)
- **Page 2, para 2:** “citability is addressed by assigning a PID.” This is not true, as the authors discuss on page 4, para 4. Indeed, page 4, para 4 seems to contradict itself. Citation is more than a locator/identifier
- In the discussion of “Data independent of any paper” it is worth noting that there may often be linkages between these data and myriad papers. Indeed a looser concept of a data paper has existed for some time, where researchers request a citation to a paper even though it is not the data nor fully describes the data (e.g the CRU temp records)
- **Page 4, para 1: **I’m not sure it’s entirely true that published data cannot involve requesting permission. In past work with Indigenous knowledge holders, they were willing to publish summary data and then provide the details when satisfied the use was appropriate and not exploitive. I think those data were “published” as best they could be. A nit, perhaps, but it highlights that there are few if any hard and fast rules about data publication.
- **Page 4, para 2:** You may also want to mention the WDS certification effort, which is combining with the DSA via an RDA Working Group:
- **Page 4, para 2:** The joint declaration of data citation principles involved many more organizations than Force11, CODATA, and DCC. Please credit them all (maybe in a footnote). The glory of the effort was that it was truly a *joint* effort across many groups. There is no leader. Force11 was primarily a convener.
- **Page 4, para 6: **The deep citation approach recommended by ESIP is not to just to list variables or a range of data. It is to identify a “structural index” for the data and to use this to reference subsets. In Earth science this structural index is often space and time, but many other indices are possible--location in a gene sequence, file type, variable, bandwidth, viewing angle, etc. It is not just for “straightforward” data sets.
- **Page 5, para 5:** I take issue with the statement that few repositories provide scientific review. I can think of a couple dozen that do just off the top of my head, and I bet most domain repositories have some level of science review. The “scientists” may not always be in house, but the repository is a team facilitator. See my general comments.
- **Page 5, para 10:** The PDS system is only unusual in that it is well documented and advertised. As mentioned, this team style approach is actually fairly common
- **Page 6, para 3:** Parsons and Fox don’t just argue that the data publication metaphor is limiting. They also say it is misleading. That should be acknowledged at least, if not actively grappled with.

We have read this submission. We believe that we have an appropriate level of expertise to confirm that it is of an acceptable scientific standard, however we have significant reservations, as outlined above.

# Response

**Published:** 2014-05-12

**Authors:** John Kratz

**Affiliations:** California Digital Library, USA

Thank you for refereeing our paper and thank you especially for delivering your report so quickly.

We submitted the paper as a review article, not an opinion piece, and it was reclassified somewhere along the way.  I contacted an editor at F1000 about the issue, and I believe it will be switched back shortly.  While there is undoubtedly a viewpoint inherent in the way we have organized the manuscript, it was our intention to deliver a timely summary of the current landscape as a foundation for future thinking, not to offer prescriptions or to endorse particular approaches.  We have no shortage of opinions about data publication, and a true opinion piece may follow at some point, but our aim here was to remain fairly neutral.  I think the paper you are asking for would also be valuable, but it's an entirely different paper from the one we have written.

That said, your report is full of suggestions for expansion of analysis and clarification of scope that would absolutely improve the paper (e.g. the question of why some issues resist consensus more than others is an excellent one), and we will certainly address them in the next version.
